# Xpro®1595 alleviates neuropathic pain by targeting spinal dorsal horn ADAM17-mediated inflammation^☆^

**DOI:** 10.1016/j.ynpai.2026.100210

**Published:** 2026-02-12

**Authors:** Li Li, Yidie Su, Ling-ling Sun, Wei-Wei Yao, Yan-Yan Sun

**Affiliations:** a Department of Anesthesiology, Huashan Hospital, Fudan University, Shanghai 200040, China; b Department of Anesthesiology, Shenzhen University General Hospital, Shenzhen University, Shenzhen, Guangdong Province 518055, China

**Keywords:** Neuropathic pain, ADAM17, A Disintegrin and A Metalloprotease17, SNL, Spared Nerve Ligation, DRG, Dorsal Root Ganglia, SDH, Spinal Dorsal Horn

## Abstract

A disintegrin and metalloprotease 17 (ADAM17) mediates the shedding of key pro-inflammatory cytokines, yet its specific contribution to neuropathic pain remains elusive. Here, we investigated the role of ADAM17 in the rat spinal nerve ligation (SNL) model. Following nerve injury, ADAM17 expression was significantly upregulated in the spinal dorsal horn (SDH) and dorsal root ganglion (DRG). Specifically, ADAM17 colocalized with TRPV1 and IB4 positive afferents in the superficial SDH, and with IB4, CGRP, and TRPV1 positive neurons in the DRG. Intrathecal administration of exogenous ADAM17 to naive rats recapitulated neuropathic pain behaviors—inducing mechanical and thermal hypersensitivity—and significantly increased the levels of TNF-α, IL-1β, and IL-6 in the SDH. Conversely, therapeutic treatment with Xpro®1595 markedly attenuated SNL-induced pain behaviors. This analgesic effect correlated with the suppression of injury-induced ADAM17 upregulation and a consequent reduction in proinflammatory cytokines. These findings demonstrate that ADAM17 is a critical driver of the neuroinflammatory cascade in neuropathic pain. Moreover, our data suggest that the analgesic efficacy of Xpro®1595 is mediated, at least in part, by disrupting this ADAM17-dependent inflammatory feedback loop.

## Highlights

• Nerve injury upregulates ADAM17 in spinal dorsal horn and DRG nociceptors.
• Intrathecal ADAM17 induces pain hypersensitivity and neuroinflammation.
• Xpro®1595 attenuates neuropathic pain and inhibits spinal ADAM17 expression.
• Xpro®1595 exerts analgesia by suppressing the ADAM17-driven cytokine cascade.

## Graphical abstract

**Figure:**
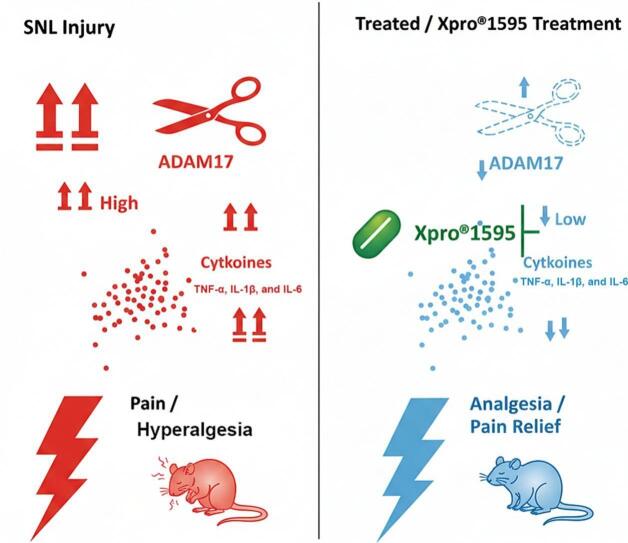

## 1 Introduction

Neuropathic pain, a debilitating chronic condition arising from lesions or diseases affecting the somatosensory nervous system, imposes a substantial burden on approximately 7–10% of the global population (Raja et al., 2020, Haanpää et al., 2011). The underlying pathophysiology is intricate, characterized by peripheral and central sensitization, neuroinflammation, and maladaptive synaptic plasticity. Central to the initiation of this pathological cascade are nociceptors, the specialized high-threshold primary sensory neurons responsible for detecting noxious stimuli. Following nerve injury, these neurons undergo profound transcriptional and functional changes, resulting in hyperexcitability and the transmission of ectopic signals to the spinal cord. Despite this understanding, current pharmacological interventions—primarily repurposed antidepressants (e.g., SNRIs, tricyclic antidepressants) and anticonvulsants (e.g., gabapentinoids)—provide meaningful relief to fewer than 30% of patients (Finnerup et al., 2021, Bidve et al., 2020). Moreover, their clinical utility is frequently compromised by dose-limiting systemic side effects, such as cognitive impairment and sedation. This unmet medical need has catalyzed the search for novel therapeutic targets with greater specificity, particularly those capable of addressing the upstream drivers of neuroinflammation.

A pivotal orchestrator of the neuroinflammatory response in neuropathic pain is the metalloproteinase family, specifically “a disintegrin and metalloproteinase 17” (ADAM17), also known as tumor necrosis factor-α converting enzyme (TACE) (Fangfang et al., 2024). ADAM17 functions as a master sheddase, governing the ectodomain cleavage and bioactivity of numerous membrane-tethered proteins. Its canonical role involves the proteolytic cleavage of transmembrane TNF-α (tmTNF) to release its soluble, pro-inflammatory counterpart, soluble TNF-α (solTNF) (Zunke and Rose-John, 2017). However, the substrate repertoire of ADAM17 extends far beyond TNF-α; it includes the IL-6 receptor, Notch receptors, and ligands for the epidermal growth factor receptor (EGFR), all of which are critical for neuronal plasticity and glial activation (Shin et al., 2022, Fredrickx et al., 2020). While the multifaceted roles of ADAM17 are well-documented in systemic immunity, oncology, and neurodegenerative disorders such as Alzheimer’s disease (Gnosa et al., 2022, Qian et al., 2016), its specific contribution to the induction and maintenance of neuropathic pain—particularly within the primary sensory neurons and the spinal dorsal horn—remains a critical gap in our understanding.

The dichotomous signaling of TNF-α presents both a therapeutic challenge and an opportunity. SolTNF predominantly binds to TNF receptor 1 (TNFR1) to mediate apoptosis and inflammation, whereas tmTNF preferentially engages TNF receptor 2 (TNFR2), promoting cell survival, immune regulation, and oligodendrocyte-mediated remyelination (Steed et al., 2003). Consequently, non-selective TNF inhibitors, which block both isoforms, carry risks of immunosuppression and demyelination by interfering with essential homeostatic tmTNF-TNFR2 signaling. This limitation has spurred the development of next-generation biologics like Xpro®1595, a dominant-negative protein engineered to selectively neutralize soluble TNF without affecting transmembrane TNF signaling (Zalevsky et al., 2007). Although preclinical studies have demonstrated the efficacy of Xpro®1595 in various CNS disorders, including male-specific reversal of mechanical allodynia (Del Rivero et al., 2019), it remains to be elucidated whether targeting downstream solTNF affects the upstream enzymatic driver, ADAM17, within the pain neuraxis.

Therefore, we hypothesized that the aberrant activation of ADAM17 within the dorsal root ganglia (DRG) and spinal dorsal horn serves as a critical driver of neuropathic pain following peripheral nerve injury. To test this, we characterized the spatiotemporal expression of ADAM17 in a rat model of spinal nerve ligation (SNL) and investigated whether the analgesic efficacy of the selective solTNF inhibitor, Xpro®1595, involves the modulation of ADAM17 expression. Collectively, this study aims to validate ADAM17 as a key upstream therapeutic target and provide a mechanistic rationale for the clinical application of selective solTNF inhibition in the management of neuropathic pain.

## 2 Materials and methods

### 2.1 Animals

Adult male and female Sprague-Dawley rats (180–220 g) were obtained from Lingchang Biotech Co., Ltd. (Shanghai, China). Animals were housed in a temperature-controlled facility on a 12-h light/dark cycle with ad libitum access to food and water. Rats were acclimatized to the laboratory environment for a minimum of 3 days prior to any experimental procedures. All protocols were approved by the Institutional Animal Care and Use Committee of Fudan University and were conducted in strict accordance with the ARRIVE guidelines and the ethical guidelines of the International Association for the Study of Pain (IASP).

### 2.2 Experimental design and drug administration

Rats were randomly allocated to experimental groups using a computer-generated randomization sequence.

Experiment 1: Effect of exogenous ADAM17 on nociception.

**Fig. 1:**
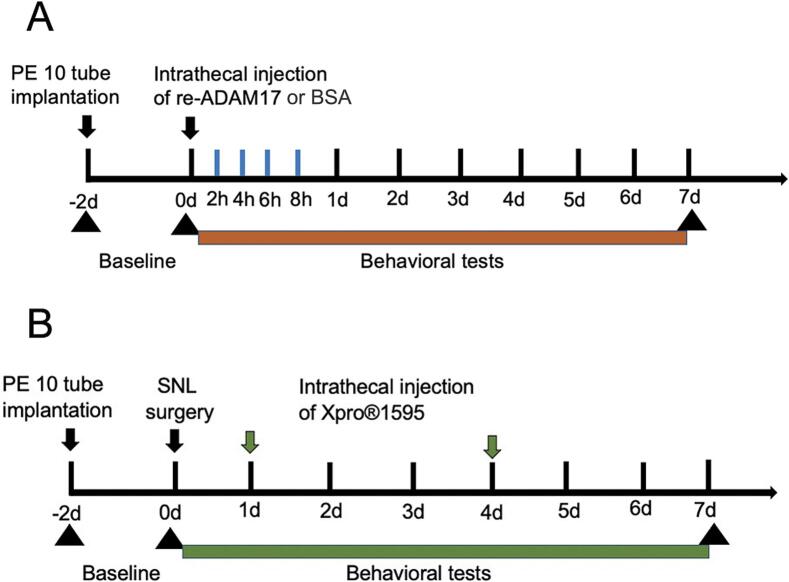
Schematic of the experimental timelines. (A) Timeline for behavioral testing following a single intrathecal injection of recombinant ADAM17 (re-ADAM17) or bovine serum albumin (BSA) vehicle in naïve rats. (B) Timeline for behavioral testing and tissue collection in spinal nerve ligation (SNL) rats following repeated intrathecal administration of Xpro®1595 or saline vehicle. Arrows indicate injection time points.

To assess the direct pronociceptive effects of ADAM17 and validate the mechanism across sexes, both male and female rats (n = 6/group/sex) were used. They received a single intrathecal (i.t.) injection of either recombinant ADAM17 (re-ADAM17) or bovine serum albumin (BSA) following catheter implantation. and behavioral assessments were conducted at the time points indicated in Fig. 1A**.**

Experiment 2: Dose-dependent efficacy of Xpro®1595.

To determine the dose-dependent efficacy of Xpro®1595, SNL-operated rats were randomly divided into five groups (n = 6/group). Male rats were used for this specific experimental series due to the restricted availability of the Xpro®1595 compound. The groups included: (1) Sham-operated control; (2) SNL + saline; and (3–5) SNL + Xpro®1595 (5, 7.5, or 10 μg/kg). Drug treatments were administered as a single bolus injection on Day 1,3 post-SNL, and behavioral assessments were conducted at the time points indicated in Fig. 1B**.**

Experiment 3: Expression and Localization.

To characterize the upregulation of ADAM17, tissues from both male and female rats were analyzed by immunofluorescence and Western blot to ensure the conservation of the target mechanism.

### 2.3 Intrathecal catheterization and spinal nerve ligation (SNL) model

Intrathecal Catheterization: Under isoflurane anesthesia, a polyethylene catheter (PE-10; I.D. 0.28 mm, O.D. 0.61 mm) was inserted into the subarachnoid space through the atlanto-occipital membrane and advanced caudally to the lumbar enlargement (L4–L5 level). The external portion of the catheter was secured in the cervical region. Rats were allowed to recover for 2 days before further procedures. Only animals displaying no motor deficits were included in the study.

Spinal Nerve Ligation (SNL) Model: The SNL model was established according to the method of Kim and Chung (1992) (Kim and Chung, 1992), with minor modifications involving single L5 nerve ligation. Following the successful implantation of the intrathecal catheter (as described above), rats were re-anesthetized with isoflurane. To minimize surgical trauma, the previous dorsal incision used for catheterization was carefully reopened and extended. The left paraspinal muscles were separated deep to the vertebral column to expose the L6 transverse process, which was then carefully removed with a small rongeur to clearly visualize the L4 and L5 spinal nerves. The left L5 spinal nerve was gently isolated and tightly ligated with two loops of 6–0 silk suture at the distal end of the dorsal root ganglion, taking great care to avoid damaging the adjacent L4 spinal nerve. The surgical site was irrigated with sterile saline, and the muscle and skin layers were sutured in layers. Local penicillin was applied to prevent infection. After recovery from anesthesia, rats were returned to their home cages under standard housing conditions. Sham-operated rats underwent the same procedure, including the reopening of the incision and removal of the L6 transverse process, but without nerve ligation.

### 2.4 Drugs and reagents

Xpro®1595 (Xencor Corporation, Monrovia, CA, USA) was dissolved in sterile saline to working concentrations of 0.1, 0.15, and 0.2 μg/μL (Bedrosian et al., 2013). Intrathecal injections were performed via the implanted catheter using a Hamilton syringe. Each rat received a volume of 10 μL followed by a 10 μL saline flush to account for the catheter dead space.

Recombinant ADAM17 (re-ADAM17; Cat# 80350-R08H, Sino Biological, Beijing, China) was dissolved in 0.01 M PBS (pH 7.4) containing 0.1% BSA and 0.1% sodium azide to prevent non-specific binding and contamination. For Experiment 1, re-ADAM17 was administered as a single 10 µL intrathecal injection.

### 2.5 Behavioral testing

All behavioral assessments were performed by an investigator blinded to the experimental groups.

#### 2.5.1 Mechanical allodynia

Mechanical sensitivity was assessed using the up-down method (Chaplan et al., 1994). Rats were acclimatized in individual Plexiglas chambers on an elevated wire mesh floor for 30 min. A series of calibrated von Frey filaments were applied to the plantar surface of the ipsilateral hind paw to determine the 50% paw withdrawal threshold (PWT).

#### 2.5.2 Thermal hyperalgesia

Thermal sensitivity was assessed using the Hargreaves plantar test (Tiwari et al., 2020). Rats were placed on a heated glass floor, and a radiant heat source was focused on the plantar surface of the hind paw. The paw withdrawal latency (PWL) was recorded with a cut-off time of 30 s to prevent tissue damage. The average of three measurements, separated by 5-minute intervals, was calculated.

### 2.6 Immunofluorescence

At designated time points, rats were deeply anesthetized and perfused transcardially with PBS followed by 4% paraformaldehyde (PFA). The L5 spinal cord segments and corresponding DRGs were harvested, post-fixed in 4% PFA, and cryoprotected in 30% sucrose. Transverse sections (25 µm thick) were cut on a cryostat. Sections were blocked and incubated overnight at 4°C with the following primary antibodies: rabbit anti-ADAM17 (ab39162, Abcam), mouse anti-ADAM17(MA565986, Invitrogen), rabbit anti-CGRP (Dia Sorin), rabbit anti-TRPV1 (ab305299, Abcam), and FITC conjugated IB4 (L2895, Sigma). After washing, sections were incubated for 2 h at room temperature with appropriate Alexa Fluor-conjugated secondary antibodies. Images were acquired using an Olympus FV1000 confocal microscope.

### 2.7 Reverse Transcription-PCR (RT-PCR)

Total RNA was extracted from L5 DRG tissues, and 2 μg of RNA was reverse-transcribed using a Reverse Transcription System (Promega, Madison, WI, USA). PCR amplification was performed using the following primers: *Adam17* (Forward: 5‘-CGCATTATCAAGCCGTTCC-3′; Reverse: 3‘-TGCTATCAACTCGGCTCT-5′) and GAPDH (Forward: 5‘-TGCTCTCTGCTCCTCCCTGTTC-3′; Reverse: 3‘-CGTTCACACCGACCTTCACCATC-5′). The cycling conditions were: 95°C for 5 min, followed by 40 cycles of 95°C for 30 s, 58°C for 30 s, and 68°C for 30 s. PCR products were resolved on a 1% agarose gel and visualized under UV light.

### 2.8 Western blot analysis

Tissues were homogenized in lysis buffer, and protein concentrations were determined. Samples were boiled at 99°C for 5 min, separated by 10% SDS-PAGE, and transferred onto PVDF membranes. Membranes were blocked with 3% non-fat milk in TBS-T for 1 h and incubated overnight at 4°C with primary antibodies against ADAM17 (Abcam, ab39162), TNF-α, IL-1β, IL-6 (Santa Cruz Biotechnology), and β-actin (Sigma). Membranes were then incubated with HRP-conjugated secondary antibodies for 2 h at room temperature. Protein bands were visualized using enhanced chemiluminescence (ECL) and quantified using Quantity One software.

### 2.9 Enzyme-Linked Immunosorbent Assay (ELISA)

Spinal cord tissues were weighed and homogenized in ice-cold lysis buffer (1:20–1:50 w/v). Homogenates were sonicated until clear and centrifuged at 10,000 × g for 5 min at 4°C. Supernatants were collected for immediate analysis or stored at −80°C. Concentrations of IL-1β, IL-6, and TNF-α were determined using rat-specific ELISA kits (Cloud-Clone Corp., China) according to the manufacturer’s instructions.

### 2.10 Statistical analysis

Data are presented as mean ± SEM. Statistical analyses were performed using GraphPad Prism 10.0. Behavioral data were analyzed using two-way repeated measures ANOVA followed by Bonferroni’s or Sidak’s post-hoc tests. Biochemical data (Western blot, ELISA, PCR) were analyzed using one-way ANOVA followed by Tukey’s or Dunnett’s post-hoc tests. A p-value < 0.05 was considered statistically significant.

## 3 Results

### 3.1 ADAM17 is upregulated in the spinal dorsal horn and colocalizes with nociceptive afferent terminals following SNL

**Fig. 2:**
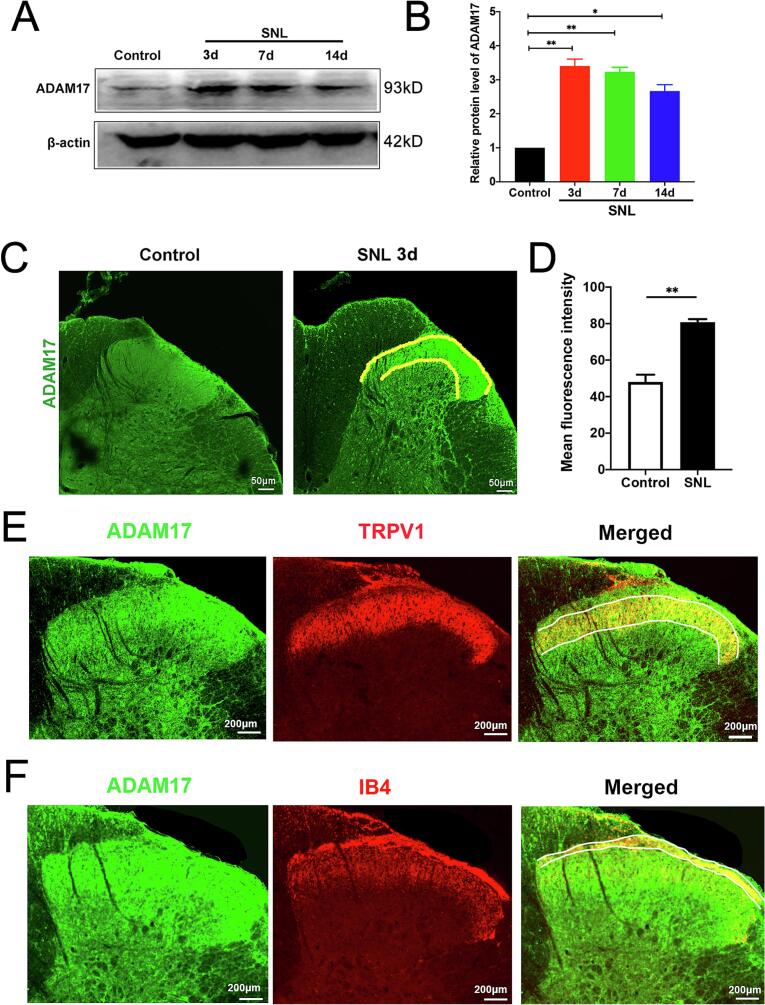
SNL induces ADAM17 upregulation in the superficial spinal dorsal horn and its colocalization with nociceptive afferent terminals. (A) Representative Western blot bands showing ADAM17 and β-actin (loading control) protein levels in the ipsilateral L4-L6 spinal dorsal horn (SDH) at different time points post-SNL or sham surgery. (B) Densitometric quantification of ADAM17 protein expression relative to β-actin. *p < 0.05, **p < 0.01. (C) Representative immunofluorescence images of ADAM17 (green) in the superficial dorsal horn (laminae I-II) at 7 days post-SNL or sham surgery. Scale bar = 50 μm. (D) Quantification of mean fluorescence intensity of ADAM17 in the superficial dorsal horn. Data are presented as mean ± SEM. n = 6 rats/group. **p < 0.01 vs. sham group. (E) Representative double-label immunofluorescence images showing the co-localization of ADAM17 (green) with TRPV1 (red) in the superficial dorsal horn (lamina II) of SNL rats at day 7. Scale bar = 200 μm. (F) Representative double-label immunofluorescence images showing the co-localization of ADAM17 (green) and IB4 (red) in the superficial dorsal horn of SNL rats at day 7 (lamina I). Scale bar = 200 μm. (For interpretation of the references to colour in this figure legend, the reader is referred to the web version of this article.)

To elucidate the role of ADAM17 in neuropathic pain, we first investigated its expression in the spinal dorsal horn (SDH) following spinal nerve ligation (SNL). Western blot analysis revealed that ADAM17 protein levels were significantly increased in the SDH of the SNL group compared to the sham-operated control group, and this upregulation was sustained for at least 14 days post-surgery **(**Fig. 2A, B**)**. Consistent with these findings, immunofluorescence staining demonstrated a marked increase in ADAM17 immunoreactivity within the superficial laminae of the ipsilateral dorsal horn—a region critical for nociceptive processing (Tsuda et al., 2017)—at 7 days post-surgery **(**Fig. 2C, D).

Small-diameter dorsal root ganglion (DRG) neurons, the primary nociceptors responsible for transmitting pain signals, are largely classified into peptidergic (TRPV1-positive) and non-peptidergic (IB4-binding) subpopulations. To determine whether the upregulated ADAM17 in the SDH was localized to the central terminals of these nociceptors, we performed double-labeling immunofluorescence. The results indicated that ADAM17 extensively colocalized with TRPV1 **(**Fig. 2E**)** and IB4-positive afferent terminals **(**Fig. 2F**)**. Collectively, these data suggest that SNL induces ADAM17 upregulation in specific nociceptive afferents within the SDH, which may contribute to maladaptive synaptic transmission and central sensitization.

### 3.2 SNL injury induces ADAM17 upregulation in peptidergic and non-peptidergic DRG neurons

**Fig. 3:**
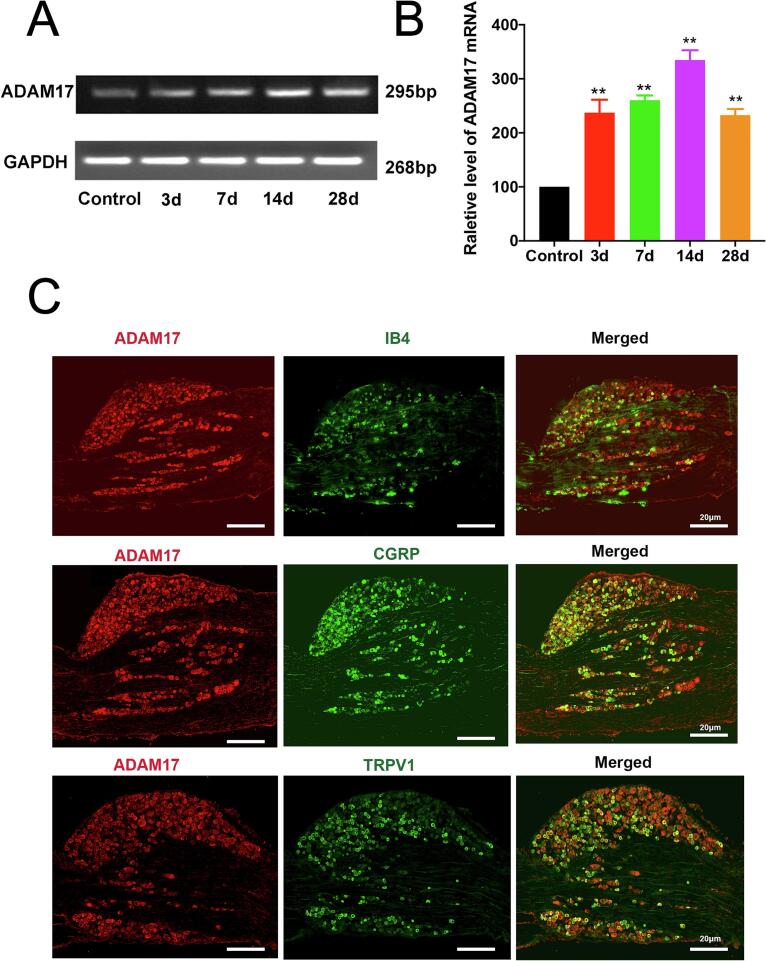
SNL induces ADAM17 upregulation in dorsal root ganglion neurons and its expression across peptidergic and non-peptidergic nociceptors. (A) Representative agarose gel images of RT-PCR products showing *Adam17* mRNA levels in the ipsilateral L5 DRG at various time points post-SNL or sham surgery. GAPDH was used as an internal control. (B) Quantification of *Adam17* mRNA levels relative to GAPDH. (C) Representative immunofluorescence images showing the co-localization of ADAM17 (red) with IB4, CGRP, or TRPV1 (green) in DRG neurons at day 14 post-SNL. Scale bar = 20 μm. Data are presented as mean ± SEM. n = 5 rats/group. **p < 0.01 vs. Sham group; One-way ANOVA followed by Tukey’s post hoc test. (For interpretation of the references to colour in this figure legend, the reader is referred to the web version of this article.)

Given that the DRG contains the cell bodies of primary sensory neurons, we next assessed ADAM17 expression in this tissue. Quantitative RT-PCR analysis showed that *Adam*17 mRNA expression in the ipsilateral L5 DRG began to increase at day 3, peaked at day 14, and remained elevated for up to 28 days following SNL **(**Fig. 3A, B**)**. To identify the specific cell types expressing ADAM17, we performed double-immunofluorescence staining. At day 14 post-SNL, we observed extensive colocalization of ADAM17 with markers of both peptidergic (CGRP, TRPV1) and non-peptidergic (IB4) nociceptors **(**Fig. 3C**)**. These results demonstrate that ADAM17 is broadly expressed across nociceptor subpopulations, providing a morphological basis for its role in pain processing. This observation aligns with a recent report showing that ADAM17 depletion specifically affects the IB4-positive subpopulation (Quarta et al., 2019). Collectively, the robust upregulation of ADAM17 in both the SDH and DRG likely represents a key molecular mechanism underlying the initiation and maintenance of neuropathic pain.

### 3.3 Exogenous ADAM17 induces pain-like behaviors and spinal neuroinflammation in naïve rats

**Fig. 4:**
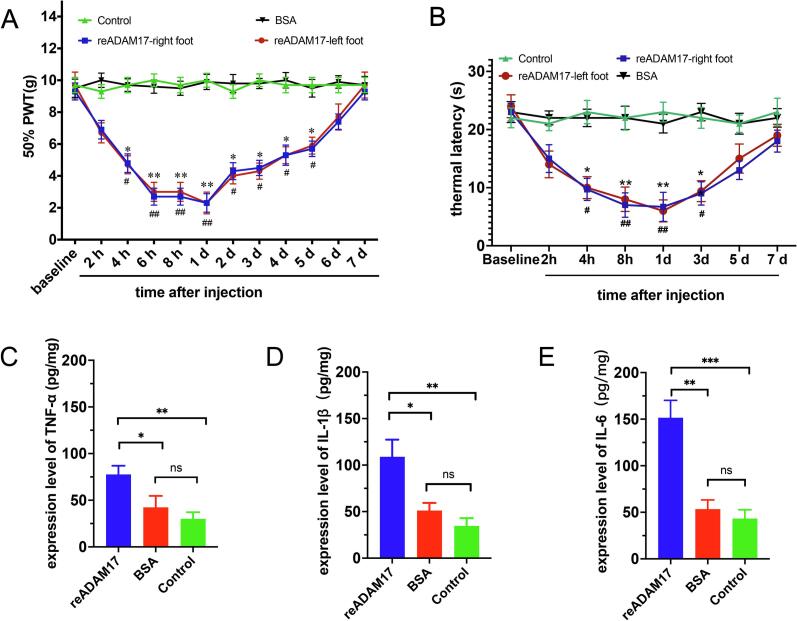
Intrathecal administration of recombinant ADAM17 induces pain-like behaviors and spinal inflammation in naïve rats. (A) Paw withdrawal threshold (PWT) in response to mechanical von Frey stimulation. (B) paw withdrawal latency (PWL) in response to radiant heat stimulation at baseline (BL) and various time points following a single intrathecal injection of recombinant ADAM17 (re-ADAM17, 0.3 μg) or BSA vehicle. Data are presented as mean ± SEM. n = 8 rats/group. *p < 0.05, **p < 0.01 reADAM 17-right vs. BSA group; ^#^*p* < 0.05, ^##^*p* < 0.01 reADAM 17-left vs. BSA group; Two-way repeated measures ANOVA followed by Bonferroni’s post hoc test. (C-E) ELISA quantification of TNF-α (C), IL-1β (D), and IL-6 (E) protein concentrations in the spinal dorsal horn 24 h post-injection. Data are presented as mean ± SEM. n = 6 rats/group. *p < 0.05, **p < 0.01, ***p < 0.001vs. BSA or Control group.

To determine whether ADAM17 upregulation is sufficient to drive pain hypersensitivity, we administered recombinant ADAM17 (re-ADAM17, 0.3 μg) via intrathecal injection to naïve rats. This single injection induced a progressive and significant decrease in the paw withdrawal threshold (PWT), indicative of mechanical allodynia, which persisted for approximately 7 days **(**Fig. 4A**)**. Similarly, treatment resulted in a significant decrease in thermal withdrawal latency (thermal hyperalgesia), which lasted for 3 days before returning to baseline by day 7 **(**Fig. 4B**)**. These results are consistent with findings that ADAM17ex/ex mice exhibit higher mechanical withdrawal thresholds compared to wild-type ADAM17+/+ mice, suggesting a pro-nociceptive role for ADAM17 (Quarta et al., 2019).

Furthermore, 24 h post-administration, re-ADAM17 significantly elevated the protein levels of TNF-α, IL-1β, and IL-6 in the spinal dorsal horn **(**Fig. 4C–E**)**. A similar increasing trend was observed in the DRG; however, likely due to the acute timeframe, these changes did not reach statistical significance (data not shown). Taken together, these data indicate that ADAM17 activity is sufficient to recapitulate neuropathic pain behaviors and trigger neuroinflammation in the absence of nerve injury.

### 3.4 Inhibition of soluble TNF-α with Xpro®1595 alleviates mechanical allodynia in SNL rats

**Fig. 5:**
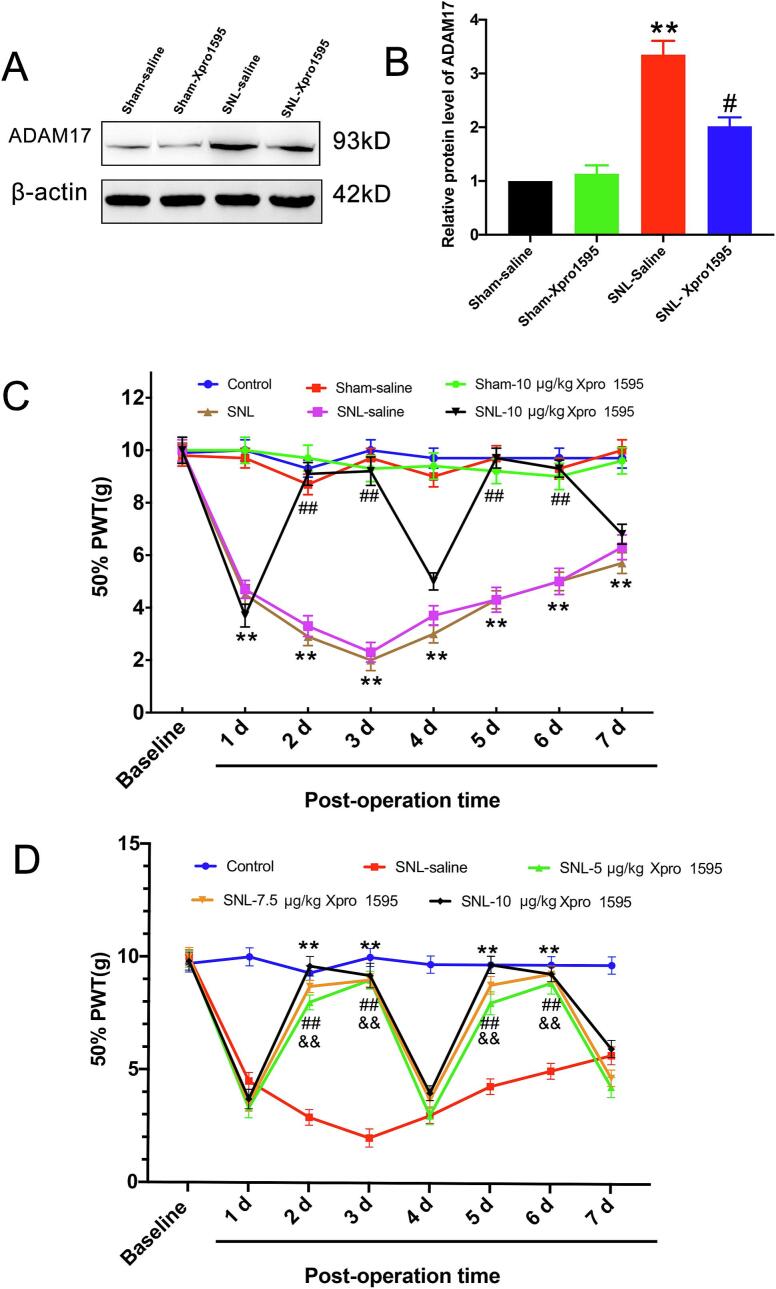
Intrathecal administration of Xpro®1595 attenuates SNL-induced mechanical allodynia and ADAM17 upregulation. (A) Representative Western blot bands showing ADAM17 protein levels in the ipsilateral SDH of sham or SNL rats treated with saline or Xpro®1595 (10 μg/kg) on day 3 post-surgery. (B) Densitometric quantification of ADAM17 protein expression relative to β-actin. (C)Mechanical paw withdrawal thresholds (PWT) in SNL rats following repeated intrathecal injections of Xpro®1595 (10 μg/kg) or saline. Arrows indicate injection time points. (D) Dose-dependent effect of Xpro®1595 (5, 7.5, or 10 μg/kg) on mechanical thresholds at day 5 post-SNL (24 h after the second injection). Data are presented as mean ± SEM. n = 6–8 rats/group. **p < 0.01 vs. sham group; #*p* < 0.05 vs. SNL-saline group; One-way ANOVA followed by Tukey’s post hoc test.

Given that a canonical function of ADAM17 is the shedding of soluble TNF-α (solTNF), we hypothesized that blocking solTNF signaling would ameliorate neuropathic pain. To test this, SNL rats were treated with Xpro®1595, a selective solTNF inhibitor. Notably, intrathecal administration of Xpro®1595 (10 μg/kg) on day 3 post-SNL significantly attenuated the injury-induced upregulation of ADAM17 protein in the SDH **(**Fig. 5A, B**)**. In behavioral assessments, a single injection of Xpro®1595 (10 μg/kg) significantly increased the 50% PWT compared to the vehicle-treated SNL group, with the analgesic effect lasting for two days. A subsequent injection on day 4 extended this therapeutic effect through day 6 **(**Fig. 5C**)**. Dose-response analysis revealed that 10 μg/kg produced a significantly greater analgesic effect than 5 μg/kg on day 5. The effect of the 7.5 μg/kg dose was intermediate **(**Fig. 5D**)**. Collectively, these findings indicate that blockade of solTNF with Xpro®1595 effectively alleviates mechanical allodynia in a dose-dependent manner, implicating the ADAM17/solTNF axis in the maintenance of neuropathic pain.

### 3.5 Xpro®1595 attenuates the SNL-induced pro-inflammatory cytokine response in the spinal cord

**Fig. 6:**
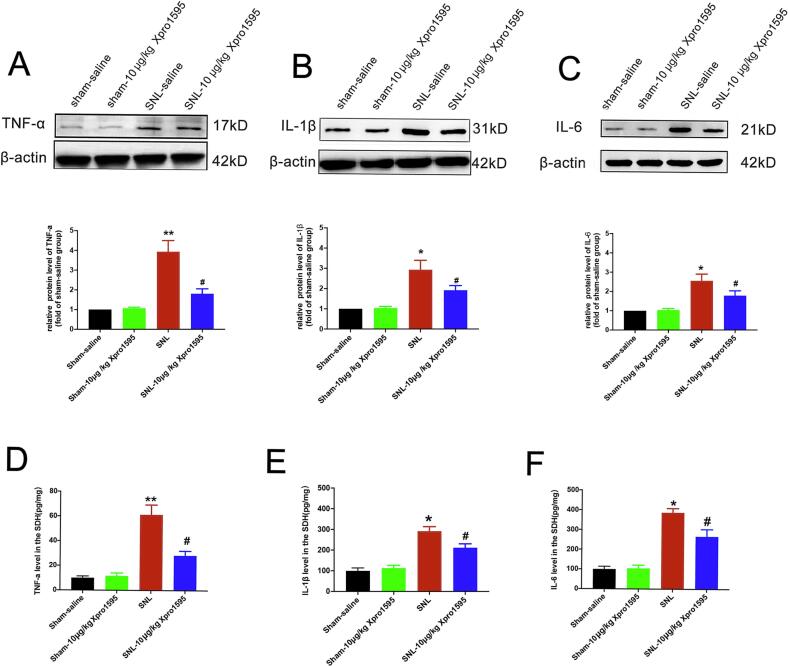
Xpro®1595 suppresses the SNL-induced pro-inflammatory cytokine response in the spinal dorsal horn. (A-C) Representative Western blot bands and corresponding quantification of TNF-α, IL-1β, and IL-6 protein levels in the ipsilateral SDH from sham, SNL + saline, or SNL + Xpro®1595 (10 μg/kg) treated rats at day 3 post-surgery. (D-F) ELISA quantification of TNF-α, IL-1β, and IL-6 protein concentrations in SDH tissue lysates from the same experimental groups. Data are presented as mean ± SEM. n = 6 rats/group. *p < 0.05, **p < 0.01 vs. sham group; ^#^*p* < 0.05, ^##^*p* < 0.01 vs. SNL + saline group; One-way ANOVA followed by Tukey’s post hoc test.

To investigate the downstream mechanisms of solTNF blockade, we measured pro-inflammatory cytokine levels in the SDH. Consistent with ADAM17 upregulation, SNL surgery resulted in significantly increased levels of TNF-α, IL-1β, and IL-6 compared to the sham group. Treatment with Xpro®1595 (10 μg/kg) robustly suppressed this injury-induced surge in all three cytokines, as determined by Western blot **(**Fig. 6A–C). These findings were corroborated by ELISA, which confirmed that protein concentrations of TNF-α, IL-1β, and IL-6 in SDH lysates were significantly lower in the Xpro®1595-treated group compared to vehicle controls (Fig. 6D–F). Thus, the analgesic efficacy of Xpro®1595 is strongly associated with the suppression of the spinal pro-inflammatory cascade, highlighting the critical role of ADAM17-mediated neuroinflammation in neuropathic pain pathology.

## 4 Discussion

The present study provides compelling evidence for the pivotal role of the ectodomain sheddase ADAM17 in the pathogenesis of neuropathic pain following peripheral nerve injury. Our primary findings demonstrate that spinal nerve ligation (SNL) induces a robust and sustained upregulation of ADAM17 in two critical sites for nociceptive processing: the spinal dorsal horn (SDH) and the dorsal root ganglion (DRG). Crucially, this upregulation was localized predominantly within the central terminals of nociceptors and their cell bodies in the DRG, specifically co-localizing with TRPV1- and IB4-positive subpopulations. This specific cellular localization positions ADAM17 as a key presynaptic regulator at the primary afferent synapse. While glial cells, particularly microglia and astrocytes, are well-established drivers of central sensitization (Old et al., 2015, Jin et al., 2003), our data highlight a critical neuronal contribution of ADAM17 to the neuroinflammatory milieu. By shedding membrane-bound substrates from nociceptive terminals, ADAM17 likely facilitates a rapid and direct modulation of synaptic transmission and the local inflammatory environment.

A key functional insight from our study is that the upregulation of ADAM17 is not merely correlative but is sufficient to drive pain-like behaviors. Intrathecal administration of recombinant ADAM17 in naïve rats recapitulated the mechanical and thermal hypersensitivities characteristic of neuropathic pain. This “gain-of-function” evidence is complemented by our pharmacological intervention with Xpro®1595, a selective inhibitor of soluble TNF-α (solTNF). We found that neutralizing solTNF—a canonical substrate of ADAM17—effectively alleviated SNL-induced mechanical allodynia. Intriguingly, Xpro®1595 treatment also attenuated the injury-induced upregulation of ADAM17 itself. This observation suggests the existence of a maladaptive positive feedback loop, wherein ADAM17-mediated shedding of solTNF drives an inflammatory response that, in turn, promotes further ADAM17 expression. While these findings are robust, future studies employing cell-specific genetic deletion of Adam17 would be valuable to dissect the precise temporal and spatial dynamics of this enzyme's contribution to pain chronicity.

Mechanistically, our results showed that intrathecal administration of Xpro®1595 not only neutralized solTNF but also robustly suppressed the downstream upregulation of IL-1β and IL-6. This broad anti-inflammatory efficacy is likely attributable to the hierarchical role of TNF-α as a “master regulator” in the neuroinflammatory cascade. TNF-α is known to orchestrate the activation of key intracellular signaling pathways, such as NF-κB and p38 MAPK, which drive the transcriptional induction of secondary cytokines including IL-1β and IL-6 (Kawasaki et al., 2008, Leung and Cahill, 2010). By selectively severing the solTNF signaling arm, Xpro®1595 effectively interrupts this feed-forward inflammatory cycle, thereby preventing the secondary surge of downstream cytokines in the spinal dorsal horn (Clausen et al., xxxx).

The therapeutic strategy of selectively inhibiting solTNF, as validated here, aligns with a growing body of evidence implicating this specific isoform in CNS pathologies. Unlike non-selective TNF inhibitors that carry risks of demyelination and immunosuppression, selective solTNF targeting preserves the neuroprotective transmembrane TNF signaling. This approach has shown promise in preclinical models of Parkinson’s disease and Alzheimer’s disease, where it mitigates neuroinflammation and microgliosis without compromising host defense (Barnum et al., 2014). Our findings extend the relevance of this precision medicine approach to the field of chronic pain, suggesting that the ADAM17-solTNF axis represents a convergent mechanism shared across diverse neuroinflammatory conditions.

## Limitations of the study

We acknowledge that the pharmacological rescue experiments in this study were conducted exclusively in male rats. While historical literature has suggested potential sex differences in immune-mediated pain processing—particularly involving microglial signaling (Sorge et al., 2015)—our data demonstrate that the core pathological mechanism identified here (i.e., ADAM17 upregulation and subsequent cytokine release) is conserved in female rats following SNL. Although we were unable to perform behavioral testing in females due to the restricted availability of the proprietary compound Xpro®1595, the conservation of the molecular target suggests that the therapeutic benefits observed in males are likely translatable to females. Future studies are warranted to empirically verify the pharmacodynamics of solTNF inhibition in female subjects when the compound becomes accessible.

In conclusion, this study establishes ADAM17 as a critical upstream mediator of neuropathic pain, acting through its upregulation in primary sensory neurons and their spinal terminals. We demonstrate that targeting the downstream effector of ADAM17—solTNF—with the selective inhibitor Xpro®1595 is an effective therapeutic strategy for alleviating pain and dismantling the associated neuroinflammatory cascade. Given the active exploration of selective solTNF inhibition in other neurological disorders, our findings highlight the ADAM17/solTNF axis as a promising, mechanistically sound therapeutic target for the management of neuropathic pain.

## 6 Consent for publication

Not applicable.

## Authors' contributions

LL, and YY.S. designed research and interpreted the data; LL. YD.S and LL.S, performed the histological examination, YD.S.LL,S, WW.Y., performed WB and behavioral research; L.L., and LL.S, analyzed data; L.L and YY.S. wrote the paper. All authors read and approved the final manuscript.

## CRediT authorship contribution statement

**Li Li:** Writing – review & editing, Writing – original draft, Software, Resources, Project administration, Methodology, Investigation, Funding acquisition, Formal analysis, Data curation, Conceptualization. **Yidie Su:** Writing – original draft, Resources, Formal analysis, Data curation. **Ling-ling Sun:** Methodology, Investigation, Data curation. **Wei-Wei Yao:** Project administration, Data curation. **Yan-Yan Sun:** Validation, Supervision, Investigation, Funding acquisition, Formal analysis, Conceptualization.

## 5 Ethics approval and consent to participate

All animal procedures were performed in accordance with the Guidelines for Care and Use of Laboratory Animals of the Fudan University and approved by the Animal Ethics Committee of the Fudan University (permit number: SYXK(HU)2014-0030).

## Funding

This work was supported by National Natural Science Foundation (82001395 to L. L), and Basic Research Project of Shenzhen Science and Technology Innovation Commission (JCYJ20210324100206017), Shenzhen Medical Research Fund (C2401009 to YY, S).

## Declaration of competing interest

The authors declare that they have no known competing financial interests or personal relationships that could have appeared to influence the work reported in this paper.

## Data Availability

Data will be made available on request.
